# Adipokines and Associations With Incident Osteoporotic Fracture in Patients With Rheumatoid Arthritis

**DOI:** 10.1002/acr.25632

**Published:** 2025-11-29

**Authors:** Joshua F. Baker, Bryant R. England, Michael D. George, Hannah Brubeck, Brian Sauer, Aleksander Lenert, Punyasha Roul, Geoffrey M. Thiele, Ted R. Mikuls, Katherine D. Wysham

**Affiliations:** ^1^ Corporal Michael J. Crescenz VA Medical Center and University of Pennsylvania Philadelphia; ^2^ VA Nebraska‐Western Iowa Health Care System and University of Nebraska Medical Center Omaha; ^3^ University of Pennsylvania, Philadelphia, and VA Nebraska‐Western Iowa Health Care System Omaha; ^4^ Puget Sound VA Health System and University of Washington Seattle; ^5^ Salt Lake City VA Medical Center and University of Utah; ^6^ VA Iowa City Health Care System Iowa City Iowa

## Abstract

**Objective:**

We assessed whether circulating adipokines are associated with incident fractures in patients with rheumatoid arthritis (RA).

**Methods:**

Three adipokines (adiponectin, leptin, and fibroblast growth factor [FGF]‐21) were measured using banked enrollment serum from participants in a longitudinal RA cohort. Adipokine levels were dichotomized as high/low using median values. Incident osteoporotic fracture was defined based on published algorithms using diagnostic codes and confirmed by chart review. Cox proportional hazard models evaluated adipokines and incident fracture risk adjusting for age, sex, race, smoking status, body mass index (BMI), prednisone use, disease activity, comorbidity score, calendar year, osteoporosis history, and previous fracture.

**Results:**

A total of 2,527 participants were included (89% male, mean age 72 years). There were 228 incident fractures over 27,540 person‐years of follow‐up (8.3 fractures per 1,000 person‐years). After adjustment, the risk of incident fracture was increased for high levels of leptin (hazard ratio [HR] = 1.47; 95% confidence interval [CI] 1.15–1.90; *P* = 0.003), FGF‐21 [HR = 1.39; 95% CI 1.16–1.67; *P* < 0.001), and adiponectin (HR = 1.21; 95% CI 0.94–1.55), with the latter not achieving significance (*P* = 0.13). Participants who had elevated levels of all three adipokines experienced twice the risk of fracture compared with those in whom none was elevated (HR = 2.17; 95% CI 1.27–3.70; *P* = 0.005).

**Conclusion:**

Elevations in adipokines are associated with an increased risk of fracture in patients with RA, independent of other established risk factors including BMI, smoking, and prednisone use. This supports further investigation to understand whether this association is related to altered body composition or disrupted metabolic pathways.

## INTRODUCTION

People with rheumatoid arthritis (RA) are known to be at higher risk of osteoporosis and osteoporotic (OP) fracture.^1^ The cause of this increased risk of fracture is multifactorial and thought to be related to the effects of chronic inflammation on bone, reduced physical activity, excess glucocorticoid use, and altered body composition.^2^ Both low body mass index (BMI) and recent weight loss are considered risk factors for OP and fracture in a number of settings, including in RA.^2^ In part, this relationship may be due to a loss of muscle mass that may directly contribute to bone loss through the loss of mechanical loading. Changes in body composition, including loss of muscle and excess adiposity, may also contribute to a higher risk of falls.^3^ Finally, other metabolic derangements occurring in the context of systemic inflammation may have independent adverse effects on bone.^4^

**SIGNIFICANCE & INNOVATIONS**

- Adipokines were associated with a greater prevalence of osteoporotic fracture and higher rates of incident fracture among patients with rheumatoid arthritis.
- Inclusion of adipokines into prediction models modestly, but significantly, improved prediction.
- High adiponectin levels were associated with fracture only among those with nonobese body mass index.

Adipokines are fat and muscle‐secreted protein hormones that function to regulate metabolism. Adipokines are closely tied to body composition abnormalities, most notably excess total and visceral adiposity. These measures therefore act as informative metabolic indicators and may render direct effects on both appetite and energy homeostasis. For example, adiponectin is observed to be higher in people who are thin and increases with rapid weight loss.^5^, ^6^ Adipokines may therefore serve as biomarkers of metabolic health and could help to predict adverse outcomes that are commonly observed in the setting of changes in energy homeostasis, weight loss, and fluctuations in weight. Adiponectin has been associated with fracture risk among men in the general population,^7^, ^8^, ^9^, ^10^ although its direct effects on bone are unclear.^11^ Leptin, which is secreted by adipocytes and considered a satiety signal, has an unclear association with bone quality, with studies demonstrating conflicting evidence characterizing its relationship with bone density and fracture.^12^, ^13^, ^14^, ^15^ Another adipokine, fibroblast growth factor (FGF)‐21, is also considered to be an indicator of metabolic stress, and higher levels have been associated with adverse bone morphology and reduced bone strength in other settings, such as among women with anorexia.^16^

Adipokines have been shown to be disrupted in patients with RA in association with disease activity^17^ and have also been linked to poor outcomes. Elevated levels of circulating adiponectin, for example, have been associated with radiographic disease progression in RA.^18^ We also previously demonstrated that FGF‐21 was independently associated with unfavorable and deteriorating body composition as well as declining physical function in patients with RA.^19^ Thus, adipokines may serve as a proxy for underlying metabolic derangements, and their measurement may provide prognostic information for a number of adverse outcomes among patients with RA. We are not aware of previous studies evaluating associations between adipokines and the long‐term risk of fracture in patients with RA.

Understanding the link between metabolic dysregulation and fracture has the potential to inform novel therapies and improve long‐term prediction of fracture. We aimed to determine if alterations in adipokine concentrations were independently associated with incident fracture in a cohort of patients with RA. We hypothesized that higher adiponectin, higher leptin, and higher FGF‐21 levels would be associated with higher rates of incident fracture independent of BMI and other important health factors.

## METHODS

### Study setting

This is a prospective cohort study conducted in the Veterans Affairs Rheumatoid Arthritis (VARA) registry and biorepository. VARA is an ongoing national repository and multicenter RA registry that has been active for more than 20 years (initiated in 2003).^20^, ^21^, ^22^, ^23^, ^24^, ^25^, ^26^, ^27^ At the time this study was conducted, 13 VA sites had contributed data. Veterans who fulfill the 1987 American College of Rheumatology classification criteria for RA and are over 18 years of age are eligible for enrollment.^28^ Rheumatology providers at each site record clinical data at enrollment and at follow‐up visits as part of routine clinical care. Each individual site has institutional review board approval, and all study patients provided informed written consent.

### Adipokine measurements

Three prehypothesized adipokines (total adiponectin, leptin, and FGF‐21) were measured at enrollment on nonfasting samples stored at −80°C for up to 16 years using validated panels from Meso Scale Discovery (Rockville, MA), as previously described.^29^, ^30^ The assays were performed in accordance with previously defined protocols at the University of Nebraska Core Laboratory. Adipokine values were log‐adjusted to fit a normal distribution and standardized so that a one‐unit difference in the value represented a one SD difference for each analyte and the mean value was equal to zero for the population. Each individual adipokine was also dichotomized at the median value to define a high vs low serum concentration. An overall “high adipokine” score was calculated as the number of adipokines above the median value (range: 0–3).

### Osteoporosis and fracture outcomes

A chart diagnosis of osteoporosis was defined by the presence of two administrative diagnosis codes over a one‐year period preceding registry enrollment from the Corporate Data Warehouse (CDW) (VA administrative data) and categorized according to Healthcare Cost and Utilization Project–Clinical Classification Software (HCUP‐CCS). The details of this classification can be found online.^31^

We initially identified potential fractures by querying inpatient and outpatient diagnoses within the medical record based on codes used in previously published algorithms in the VA.^32^ All fractures identified using these diagnosis codes were adjudicated by a manual chart review of radiographs and medical notes from clinical providers. During chart review, we excluded fractures that were sustained during high‐velocity trauma, periprosthetic fractures, fractures of face, fingers and toes, and pathologic fractures from cancer. Fractures occurring before enrollment in the registry were considered prevalent fractures. Only the first fracture occurring after enrollment in the registry was adjudicated, and follow‐up time after the first fracture was censored in analyses.

### Bone density assessments

Results of dual X‐ray absorptiometry (DXA) testing were extracted from the registry database (entered by local investigators). Because entry of DXA results into the registry database is not uniform across all sites, we additionally queried CDW for additional results of DXA testing and extracted the corresponding radiology reports. The closest DXA result to the enrollment visit was manually reviewed for each participant, and the impression (normal, osteopenia, osteoporosis) was recorded. Bone density and T‐score data were not uniformly available from these notes.

### RA disease activity, serologies, markers of inflammation

Clinical joint counts (0 to 28) and patient/physician global scores were extracted from the registry. C‐reactive protein (CRP) (mg/dL) levels were assessed using nephelometry (Siemens, Munich, Germany). Our primary disease activity measure was the Disease Activity Score in 28 Joints with CRP (DAS28[CRP]).^33^ From banked serum, a second‐generation commercial anticyclic citrullinated peptide antibodies (ACPA) were also assessed using a second‐generation commercial assay (range 0–5 u/mL).^34^

### Other covariables

Demographics and disease‐specific characteristics were obtained from the VARA registry database. BMI was extracted from the vital sign packages available in the CDW, and the closest BMI value (within 60 days) to the enrollment date was used. BMI categories were defined as underweight (<20), normal weight (≥20–24.99), overweight (≥25–29.99), obese (≥30–34.99), and severely obese (≥35).^35^

We also defined the presence of metabolic syndrome based on the ATP III definition that requires three of the five following criteria to be present: elevated waist circumference (≥100 cm for men, ≥90 cm for women), low high‐density lipoprotein, high triglycerides, evidence of insulin resistance, and high blood pressure. Because waist circumference was not available in this sample, we used previously validated algorithms to predict waist circumference from age, sex, and BMI, as previously described.^36^ Insulin resistance was considered to be present if diabetes was present at enrollment or a Hemoglobin A1c value within 6 months of enrollment was greater than 6%.

Comorbidity was assessed at enrollment using the rheumatic disease comorbidity index (RDCI) with one point removed in the presence of fracture.^37^ Individual comorbidities were extracted over a one‐year period preceding enrollment from CDW and categorized according to HCUP‐CCS.^30^

RA treatments were extracted from VA pharmacy databases. Participants were considered to be exposed to the therapy at enrollment if the enrollment visit occurred during a defined medication course.^23^ Active glucocorticoid use was considered to be present at enrollment if a course of prednisone (not including dose‐packs) overlapped within 30 days of the enrollment visit (positive predictive value = 85%).^29^, ^30^ Previous use and recent use (within 1 year of enrollment) of a bisphosphonate therapy was extracted from pharmacy databases.

### Statistical analysis

Characteristics of the study population were described among participants who sustained or did not sustain an OP fracture in follow‐up. Missing values for DAS28(CRP) were imputed by either replacing it with the DAS28 with erythrocyte sedimentation rate (N = 183) or through conditional mean imputation (N = 242, 9.6%). Missing values for baseline BMI were also imputed (5.1%) using conditional mean imputation. Logistic regression was used to evaluate the cross‐sectional associations between individual adipokines (above/below the median; per 1 SD) with the presence of a history of osteoporosis (based on diagnostic codes and based on DXA impression) or OP fracture before enrollment after adjusting for age, sex, race, BMI, smoking status (current, former, never), DAS28, calendar year, comorbidity score, and the use of prednisone.

The primary analyses used Cox proportional hazards models to assess associations between adipokine measures and the time to incident fracture among all participants, clustering on study site and censoring at the first fracture, end of follow‐up, or death. Hypothesized confounders that were explored in the analysis included baseline demographics, BMI, smoking status, RA disease duration, RA disease activity, ACPA status (positive vs negative), RDCI, history of osteoporosis by diagnosis codes, use of osteoporosis medications within a year of enrollment, history of previous OP fracture, and calendar year. Analytes were examined separately and then together in a single model. We explored further adjustment for DXA impression at enrollment (ie, normal, osteopenia, osteoporosis). We also explored several prehypothesized interactions with age, sex, and BMI.

The proportional hazards assumption was tested by visualizing the Shoenfeld residuals for each adipokine. There was no evidence of violation of the proportional hazards assumption. The analysis was conducted with Stata v.18.0 (College Station, TX) within the VA Informatics and Computing Infrastructure.

## RESULTS

A total of 2,527 registry participants were evaluated at baseline. Enrollment characteristics of participants who did and did not experience fracture during follow‐up are shown in Table 1. At enrollment, 158 (6.3%) participants had two diagnosis codes for osteoporosis and 81 (3.2%) had experienced a previous OP fracture. In the overall sample, the median (interquartile range [IQR]) values for adiponectin, leptin, and FGF‐21 were 12.7 (6.1–25.3) ug/mL, 10.2 (3.8–23.9) ng/mL, and 0.61 (0.33–1.14) ng/mL, respectively.

**Table 1:** Participant characteristics at enrollment of those who did and did not develop a fracture in follow‐up*

Patient characteristics	Fractured, N = 228	Did not fracture, N = 2,299	*P* value
Age, mean (SD), y	64.6 (9.7)	64.2 (11.0)	0.69
Female, n (%)	44 (19)	242 (10)	<0.001
White, n (%)	184 (81)	1,765 (91)	0.18
Black, n (%)	24 (11)	374 (16)	0.02
Smoking, n (%)			
Current	55 (24)	452 (20)	0.07
Former	108 (47)	1,229 (53)	
BMI, mean (SD)	28.6 (6.4)	28.8 (5.7)	0.61
RDCI, mean (SD)	3.70 (2.0)	3.34 (2.0)	0.01
DAS28, mean (SD)	3.78 (1.37)	3.59 (1.45)	0.07
ACPA positive, n (%)	169 (74)	1,658 (72)	0.44
Disease duration, median (IQR), y	9.0 (2.8–20.8)	7.5 (2.3–16.6)	0.07
Methotrexate, n (%)	115 (50)	1,195 (52)	0.66
Glucocorticoids, n (%)	39 (45)	828 (34)	0.04
TNFi therapy, n (%)	55 (24)	584 (25)	0.67
Enrolled <2010, n (%)	132 (58)	1,144 (50)	0.02
Osteoporosis, n (%)	15 (7)	143 (6)	0.83
Previous OP fracture, n (%)	18 (8)	63 (3)	<0.001
Previous bisphosphonate, n (%)	96 (42)	612 (27)	<0.001
Diabetes, n (%)	79 (35)	755 (33)	0.58
Hypertension, n (%)	157 (69)	1,576 (69)	0.92
Heart failure, n (%)	26 (11)	230 (10)	0.50
Osteoarthritis, n (%)	191 (84)	1,840 (80)	0.18
COPD/asthma, n (%)	88 (39)	728 (32)	0.03
DXA impression, n (%)			<0.001
Normal	31 (14)	598 (26)	
Osteopenia	91 (40)	784 (34)	
Osteoporosis	56 (25)	284 (12)	
Not done/missing	50 (22)	633 (28)	
Adipokines, median (IQR)			
Adiponectin, ug/mL	14.8 (7.0–31.6)	12.5 (6.0–25.0)	0.02
Leptin, ng/mL	12.3 (4.5–28.1)	10.0 (3.8–23.3)	0.03
FGF‐21, pg/mL	721 (418–1,283)	598 (318–1,130)	0.009
Follow‐up time, y	5.4 (2.6–8.7)	12.2 (6.8–15.6)	<0.001

* ACPA, anticyclic citrullinated peptide antibody; BMI, body mass index; COPD, chronic obstructive pulmonary disease; DAS28, disease activity score in 28 joints; DXA, dual energy absorptiometry; FGF‐21, fibroblast growth factor 21; IQR, interquartile range; OP, osteoporotic; RDCI, rheumatic disease comorbidity index; TNFi, tumor necrosis factor inhibitor.

**Figure 1:**
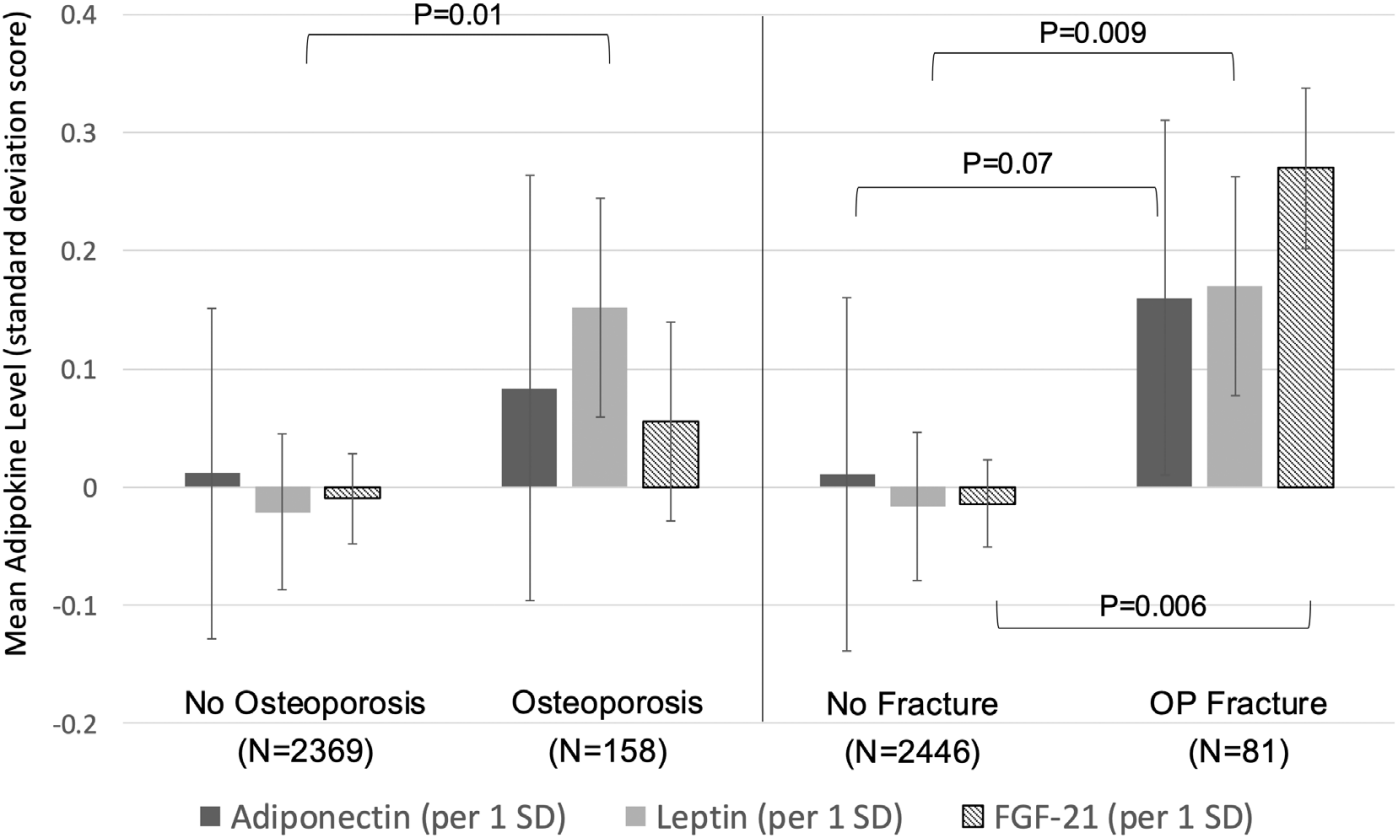
Adipokine levels by diagnosis of osteoporosis (two codes in last year) or history of previous osteoporotic fracture, at enrollment adjusted for age, sex, race, BMI, DAS28, smoking, RDCI, and prednisone use, and calendar date of enrollment. Adjusted for age, sex, race, BMI, DAS28, smoking status, RDCI, prednisone use, and calendar date. BMI, body mass index; DAS28, disease activity score in 28 joints; OP, osteoporotic; RDCI, rheumatic disease comorbidity index.

Higher levels of leptin were observed among those with a diagnosis of osteoporosis before enrollment after adjusting for age, sex, race, BMI, comorbidity score, smoking status, calendar year, DAS28, and use of prednisone (Figure 1). In addition, all adipokine levels were higher among patients with a history of OP fracture compared with those that did not have a history of previous fracture before and after adjustment (Table 1 and Figure 1).

In cross‐sectional analyses, accounting for covariables, adiponectin, and FGF‐21 levels above the median were associated with higher odds of previous/prevalent OP fracture at baseline (Table 2). Likewise, participants in whom all three adipokines were above the median had greater odds of a previous fracture compared with those in whom none was elevated (odds ratio [OR] = 2.71; 95% confidence interval (CI) 1.14–6.45; *P* = 0.03). There were no significant associations between adipokines and DXA impression at baseline after adjustment (Supplementary Table 1).

**Table 2:** Elevations in adipokines and associations with prevalent osteoporosis and fracture at enrollment adjusting for age, sex, race, BMI, prednisone use, disease activity, and smoking status*

Adipokine	Baseline osteoporosis, n = 158/2527		Previous OP fracture, n = 83/2527	
	OR (95% CI)	*P* value	OR (95% CI)	*P* value
High adiponectin	1.03 (0.74–1.42)	0.88	1.75 (1.10–2.79)	0.02
High leptin	1.36 (0.94–1.98)	0.10	1.22 (0.73–2.05)	0.44
High FGF‐21	1.15 (0.83–1.60)	0.41	1.73 (1.09–2.73)	0.02
Number elevated				
None	1 (reference)	–	1 (reference)	–
1 elevated	1.39 (0.78–2.46)	0.26	1.21 (0.53–2.76)	0.64
2 elevated	1.52 (0.78–2.96)	0.22	1.66 (0.74–3.73)	0.22
All elevated	1.45 (0.69–3.03)	0.33	2.71 (1.14–6.45)	0.03

* Adjusted for age, sex, race, BMI, DAS28, smoking, RDCI, and prednisone use, and calendar date of enrollment; the presence of prevalent osteoporosis is based on diagnostic codes present before registry enrollment. The RDCI was adjusted down one point for those with prevalent fracture. BMI, body mass index; CI, confidence interval; DAS28, disease activity score in 28 joints; FGF‐21, fibroblast growth factor‐21; OP, osteoporotic; OR, odds ratio; RDCI, rheumatic disease comorbidity index.

In longitudinal assessments, there were 228 participants with RA who developed an OP fracture over 27,540 person‐years of follow‐up, which is a rate of 8.3 fractures per 1,000 person‐years between 2003 and 2020. The median time to fracture was 5.4 years (IQR 2.6–8.6 years) and the range of follow‐up time in the overall sample was 42 days to 17.4 years. Patients who experienced a fracture during follow‐up were older and more likely to use glucocorticoids, be female, have a diagnosis of osteoporosis, and to have lower rates of normal bone density on DXA (Table 1).

Higher levels of FGF‐21 and leptin (above the median) were associated with an increased risk of incident fracture in adjusted models. For example, levels of leptin above the median were associated with a 47% higher risk of fracture [hazard ratio (HR): 1.47; 95% CI 1.15–1.90; *P* = 0.003). Leptin and FGF‐21 were both associated with fracture independent of each other (Table 3). Adiponectin levels above the median were not significantly associated with fracture risk (HR 1.21; 95% CI 0.94–1.55; *P* = 0.13).

**Table 3:** Association between adipokines and incident fracture*

Adipokine	Incident osteoporotic fracture (adipokines separately), N = 2,527, P‐Y = 16,572; events = 228		Incident osteoporotic fracture (adipokines together), N = 2,527, P‐Y = 16,572; events = 228	
	HR (95% CI)	*P* value	HR (95% CI)	*P* value
High adiponectin	1.21 (0.94–1.55)	0.13	1.17 (0.91–1.50)	0.29
High leptin	1.47 (1.15–1.90)	0.003	1.34 (1.11–1.63)	0.003
High FGF‐21	1.39 (1.16–1.67)	<0.001	1.39 (1.07–1.82)	0.02

* In the left column, the result represents the effect for the exposure in three separate models. In the right column, adipokines were tested in the same model. All models adjusted for age, age^2^, sex, race, BMI, smoking, history of osteoporosis, history of fracture, previous use of bisphosphonates, DAS28, prednisone use, comorbidity score (RDCI), and date of enrollment. The RDCI was adjusted down one point for those with prevalent fracture. BMI, body mass index; CI, confidence interval; FGF‐21, fibroblast growth factor‐21; HR, hazard ratio; P‐Y, person‐years; RDCI, rheumatic disease comorbidity index.

**Figure 2:**
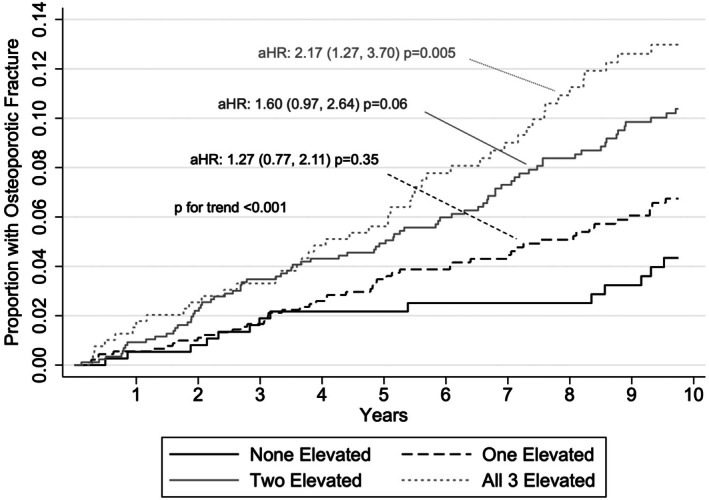
Kaplan‐Meier curve demonstrating the proportion at risk experiencing an osteoporotic fracture over time according to the number of elevated adipokines. Models are adjusted for age, sex, race, calendar year, BMI, smoking, osteoporosis at enrollment, previous osteoporotic fracture, current prednisone use, DAS28, RDCI, and previous bisphosphonate use.aHR, adjusted hazard ratio; BMI, body mass index; DAS28, disease activity score in 28 joints; RDCI, rheumatic disease comorbidity index.

Higher adipokine scores (range: 0–4) were associated with an increased fracture risk in a dose‐dependent manner (Figure 2) (*P* for trend <0.001). A high adipokine score of three (all adipokines elevated) (N = 393) was associated with a more than two‐fold increase in risk compared with a score of zero (none elevated) (N = 371) (HR 2.17; 95% CI 1.27–3.70; *P* = 0.005) (Figure 2). Adding the adipokine score to the model improved the overall prediction of the model (Harrell's C‐statistic 0.678 vs 0.664; *P* = 0.01).

Adipokines were not significantly associated with DXA impression at enrollment (Supplementary Table 1). Models further adjusting for DXA impression at enrollment also did not significantly affect the point estimates for adipokines (Supplementary Table 2). Associations between adipokines and incident fracture modeled as continuous variables or by quartile are shown in Supplementary Table 3. The presence of metabolic syndrome was not associated with fracture (HR 0.98; 95% CI 0.84–1.15; *P* = 0.81), and adding it to the model did not affect estimates for adipokines (not shown).

There was a significant interaction between adiponectin and BMI such that a higher level of adiponectin was strongly associated with fracture among those with a nonobese BMI (HR for <30: 1.52; 95% CI 1.09–2.12; *P* < 0.001; *P* for interaction <0.001) (Supplementary Table 4). There were no significant interactions between BMI and leptin or FGF‐21. There were no significant interactions with age, although high adiponectin levels tended to be associated with fracture among adults over 65 years of age (HR 1.62; 95% CI 0.94–2.80; *P* = 0.08). (Supplementary Table 5). There were no significant interactions between adipokines with sex on fracture risk.

## DISCUSSION

This study demonstrated associations between circulating adipokines and the risk of incident OP fracture among patients with RA. In particular, those with elevated levels of all measured adipokines had more than twice the risk of incident OP fracture. These observations were independent of a number of other risk factors, including demographics, BMI, smoking, glucocorticoid use, RA disease activity, and comorbidity, in addition to a previous diagnosis of osteoporosis, fracture, and bone density assessments. These findings indicate that metabolic assessments beyond clinically available measures may aid in OP fracture risk stratification in RA.

Associations between adipokines and OP fracture have been observed in other populations.^7^ For example, in the Osteoporotic Fractures in Men study, the investigators found that higher adiponectin levels (per SD) were associated with a 30% higher risk of fracture in adjusted models.^10^ In another study by Nakamura et al, high adiponectin levels were associated with fracture among postmenopausal women.^12^ Although we did not find a significant association between adiponectin and fracture overall, there was a significant association among patients who were nonobese in this study, which is a finding that is largely consistent with these previous studies. The current study may suggest that associations are stronger in older and thinner patients who may be more likely to suffer from sarcopenia or cachexia, as has previously been suggested.^38^

Nakamura et al observed that higher levels of leptin were associated with a lower rate of fracture among postmenopausal women.^12^ Other studies have found no association between leptin and fracture risk.^13^, ^39^ In addition, although some studies have identified positive relationships between leptin and bone mineral density,^14^ others have found inverse associations after considering adiposity.^15^ We are not aware of previous studies evaluating this question in RA, which is a disease that is associated with metabolic disturbances, weight loss, sarcopenia, and heightened fracture risk. These differences across studies imply that associations between leptin and FGF‐21 with fracture outcomes may be expected to vary based on the population under study and the definition for fracture used. Leptin and FGF‐21 are strongly associated with obesity, and obesity has been increasingly recognized to be a risk factor for fracture, particularly among patients that are at risk for sarcopenic obesity due to functional impairment and a greater risk of falls.^40^, ^41^, ^42^, ^43^

Adipokines are metabolic regulators that play a role in maintaining energy balance and may help predict outcomes by serving as a proxy for metabolic derangements. During chronic inflammatory disease states, large amounts of energy are expended by the activated immune system. Therefore chronic inflammation can disrupt normal energy homeostasis and result in fatigue, cachexia, anemia, poor physical function, insulin resistance, and bone loss, among other consequences.^4^ We have previously shown that adipokines are associated with muscle loss in patients with RA, which is an important risk factor in the development of low bone density and leads to an increased risk of falls and osteoporotic fracture.^44^, ^45^ Osteoblasts and osteoclasts do express adiponectin receptors, and some studies have suggested that adiponectin may have direct effects on osteoblasts and osteoclasts in vitro.^46^ However, the role of adiponectin in bone remodeling and repair is poorly understood.^11^ Further study is needed to determine if adipokines directly impact bone quality, thereby serving as potential therapeutic targets in conditions in which metabolic disruptions are often observed, as in patients with inflammatory conditions such as RA.

Adipokines have been hypothesized to be associated with fracture risk through an association with metabolic obesity and metabolic syndrome. The relationship between metabolic obesity and bone health is complex and not fully understood; however, one study demonstrated higher rates of fracture among those with high waist circumference.^47^ Although obesity is often observed to be protective against fracture, the metabolic complications of obesity may be deleterious.^48^ In our study, we found no evidence of an association between metabolic syndrome and fracture, and the association between adipokines and fracture was independent of the presence of metabolic syndrome. Thus, we did not find evidence that adipokines increase the risk of fracture through their role as markers of metabolic obesity in this population. Further study in this area may be needed.

Despite adipokines being associated with significantly increased risks of incident OP fracture, they offered only modest prognostic value. Thus, although the current study suggests that the assessment of metabolic factors may be important in understanding risks of fracture in this population, biomarkers with greater prognostic value may be needed to add significantly to clinical care. Our findings that associations of adipokines with fracture risk were independent of baseline DXA impressions suggests that peripheral biomarkers could potentially aid in risk prediction above other imaging biomarkers in common use. However, additional study is needed to generate a potential tool from these markers, generate optimal cut‐points for clinical use, and to quantify the potential benefits and the key subgroups for which its use might be most valuable. It may also be possible to combine adipokine assessments with other biomarkers to generate a more accurate and clinically‐valuable tool.

Our study includes a high proportion of men with RA from a population of US veterans and may not be completely generalizable to other RA populations, particularly since women are at higher risk of osteoporosis and fracture over their lifetimes. One previous study did find that the effect of adiponectin on fracture risk, for example, was stronger for men compared with women.^7^ Although there were no significant interactions by biologic sex in our study, this does not rule out clinically meaningful differences in association, and these observations should be validated in populations with a greater proportion of women. Although we adjusted for previous diagnosis of osteoporosis, DXA findings, and fracture history, there may be residual confounding that limits our ability to make direct causal inferences; although the results do suggest a prognostic value beyond other information typically available in clinic. It is also worth noting that fractures that may have been cared for outside of the VA might have been missed, particularly if that care was not billed to the VA. However, many of these fractures may also be captured by subsequent routine outpatient VA care.^49^ We had to impute some covariates because of missing data (eg, disease activity) within the registry, which may have limited our ability to fully adjust for these covariates. Finally, the data available did not provide an opportunity to directly compare prediction with the FRAX calculator; thus, it remains unclear whether adipokines provide value above and beyond this risk‐prediction tool. Strengths of the study include the large sample of at‐risk patients, comprehensively assessed adipokine measures and defined thresholds, thorough adjustment for comorbidity and clinical disease activity, as well as the adjudicated fracture outcome.

In summary, elevated levels of the adipokines leptin and FGF‐21 are independent predictors of incident OP fracture among patients with RA. Further study is needed to understand the clinical utility of the assessment of adipokines in the clinical management of patients with RA, as well as to clarify the mechanisms underlying this association.

## AUTHOR CONTRIBUTIONS

All authors contributed to at least one of the following manuscript preparation roles: conceptualization AND/OR methodology, software, investigation, formal analysis, data curation, visualization, and validation AND drafting or reviewing/editing the final draft. As corresponding author, Dr Baker confirms that all authors have provided the final approval of the version to be published and takes responsibility for the affirmations regarding article submission (eg, not under consideration by another journal), the integrity of the data presented, and the statements regarding compliance with institutional review board/Declaration of Helsinki requirements.

## Supporting information


**Disclosure form**.

**Supplementary Table 1:** Adjusted mean (SE) levels of adipokines by DXA category.
**Supplementary Table 2:** Association between individual adipokines and incident fracture after adjusting for DXA impression. The result represents the effect for the exposure in three separate models.
**Supplementary Table 3:** Association between adipokines (assessed as continuous variables) and incident fracture. Adipokines were tested individually in three separate models.
**Supplementary Table 4:** Association between individual adipokines and incident osteoporotic fracture stratifying by obese v. non‐obese.
**Supplementary Table 5:** Association between individual adipokines and incident osteoporotic fracture stratifying by obese v. non‐obese.
