# Supplementary material for: Adipokines and Associations With Incident Osteoporotic Fracture in Patients With Rheumatoid Arthritis
Source: Arthritis Care Res (Hoboken). 2025 Nov 29;78(3):316–24. doi: 10.1002/acr.25632 (PMC12975670; doi:10.1002/acr.25632)
Supplement: Supplementary file 2 — Supplementary Table 1: Adjusted mean (SE) levels of adipokines by DXA category. Supplementary Table 2: Association between individual adipokines and incident fracture after adjusting for DXA impression. The result represents the effect for the exposure in three separate models. Supplementary Table 3: Association between adipokines (assessed as continuous variables) and incident fracture. Adipokines were tested individually in three separate models. Supplementary Table 4: Association between individual adipokines and incident osteoporotic fracture stratifying by obese v. non‐obese. Supplementary Table 5: Association between individual adipokines and incident osteoporotic fracture stratifying by obese v. non‐obese. [file ACR-78-316-s001.docx]

**Supplementary Material**

Supplementary Table 1: Adjusted mean (SE) levels of adipokines by DXA category.

|  | Not Done or Missing | Normal  T>-1.0 | Osteopenia  T -1.0 to -2.4 | Osteoporosis  T < -2.5 |
| --- | --- | --- | --- | --- |
|  | N=683 | N=629 | N=875 | N=340 |
| Adiponectin (SD) | -0.11*  (0.038) | 0.053  (0.040) | 0.062  (0.033) | 0.089  (0.054) |
| Leptin (SD) | -0.008  (0.030) | 0.020  (0.031) | -0.024  (0.026) | -0.034  (0.043) |
| FGF-21 (SD) | 0.030  (0.038) | -0.056  (0.040) | -0.012  (0.033) | 0.036  (0.054) |

*p<0.05 compared to “Normal” bone density.

*Adjusted for age, sex, race, BMI, DAS28, calendar date, smoking, RDCI, and prednisone use.

Supplementary Table 2: Association between individual adipokines and incident fracture after adjusting for DXA impression. The result represents the effect for the exposure in three separate models.

|  | **Incident Osteoporotic Fracture**  **(Adipokines Separately)**  N=2527  P-Y=27,540; Events: 228 | |
| --- | --- | --- |
|  | HR (95% CI) | P |
| High Adiponectin | 1.17 (0.91, 1.51) | 0.23 |
|  | HR (95% CI) | P |
| High Leptin | 1.47 (1.14, 1.91) | 0.003 |
|  | HR (95% CI) | P |
| High FGF-21 | 1.37 (1.13, 1.66) | 0.001 |
|  |  |  |

All models adjusted for age, age^2^, sex, race, BMI, smoking, history of osteoporosis, history of fracture, prior use of bisphosphonates, DAS28, prednisone use, comorbidity score (RDCI), date of enrollment, and DXA impression

Note: The RDCI was adjusted down 1 point for those with prevalent fracture.

Abbreviations: P-Y= Person-years; HR= Hazard Ratio; CI= Confidence Interval; BMI= Body Mass Index; DXA= Dual Energy Absorptiometry

Supplementary Table 3: Association between adipokines (assessed as continuous variables) and incident fracture. Adipokines were tested individually in three separate models.

|  | Incident Osteoporotic Fracture  N=2527  P-Y=27,540; Events: 228 | |
| --- | --- | --- |
|  | HR (95% CI) | P |
| Adiponectin (per SD) | 1.07 (0.94, 1.22) | 0.30 |
| Adiponectin Quartile (v.1) | 1 (reference) | -- |
| 2 | 0.93 (0.71, 1.24) | 0.64 |
| 3 | 1.18 (0.85, 1.64) | 0.33 |
| 4 | 1.17 (0.77, 1.79) | 0.46 |
|  | HR (95% CI) | P |
| Leptin (per SD) | 1.10 (0.94, 1.29) | 0.23 |
| Leptin Quartile (v.1) | 1 (reference) | -- |
| 2 | 0.96 (0.74, 1.23) | 0.73 |
| 3 | 1.20 (0.86, 1.67) | 0.28 |
| 4 | 1.49 (1.05, 2.10) | 0.03 |
|  | HR (95% CI) | P |
| FGF21 (per SD) | 1.13 (0.98, 1.30) | 0.09 |
| FGF-21 Quartile (v.1) | 1 (reference | -- |
| 2 | 0.93 (0.60, 1.44) | 0.76 |
| 3 | 1.40 (1.14, 1.72) | 0.001 |
| 4 | 1.26 (1.02, 1.60) | 0.03 |
|  |  |  |

All models adjusted for age, age^2^, sex, race, BMI, smoking, history of osteoporosis, history of fracture, prior use of bisphosphonates, DAS28, prednisone use, comorbidity score (RDCI), and date of enrollment.

Note: The RDCI was adjusted down 1 point for those with prevalent fracture.

Abbreviations: P-Y= Person-years; HR= Hazard Ratio; CI= Confidence Interval; BMI= Body Mass Index; DXA= Dual Energy Absorptiometry

Supplementary Table 4: Association between individual adipokines and incident osteoporotic fracture stratifying by obese v. non-obese.

|  | Non-Obese  (Adipokines Separately)  N=1,643  P-Y= 18,224; Events: 151 | | Obese  (Adipokines Separately)  N=884  P-Y=9320; Events: 77 | |  |
| --- | --- | --- | --- | --- | --- |
|  | *HR (95% CI)* | *p* | *HR (95% CI)* | *p* | *p for interaction* |
| High Adiponectin | 1.53 (1.10, 2.11) | 0.01 | 0.75 (0.47, 1.19) | 0.22 | <0.001 |
|  | *HR (95% CI)* | *p* | *HR (95% CI)* | *p* |  |
| High Leptin | 1.58 (1.02, 2.45) | 0.04 | 1.33 (0.84, 2.12) | 0.22 | 0.41 |
|  | *HR (95% CI)* | *p* | *HR (95% CI)* | *p* |  |
| High FGF-21 | 1.27 (0.99, 1.62) | 0.06 | 1.62 (1.26, 2.07) | <0.001 | 0.71 |
|  |  |  |  |  |  |
| Adipokine Score | *HR (95% CI)* | *p* | *HR (95% CI)* |  | *p for interaction* |
| 1 | 1 (reference) | -- | 1 (reference) | -- |  |
| 2 | 1.23 (0.69, 2.10) | 0.49 | 1.30 (0.39, 4.30) | 0.67 | 0.86 |
| 3 | 1.52 (1.00, 2.33) | 0.052 | 1.65 (0.55, 4.92) | 0.37 | 0.002 |
| 4 | 2.51 1.51, 4.20) | <0.001 | 1.52 (0.64, 3.59) | 0.34 | 0.19 |

All models adjusted for age, age^2^, sex, race, BMI, smoking, history of osteoporosis, history of fracture, prior use of bisphosphonates, DAS28, prednisone use, comorbidity score (RDCI), and date of enrollment.

Note: The RDCI was adjusted down 1 point for those with prevalent fracture.

Supplementary Table 5: Association between individual adipokines and incident osteoporotic fracture stratifying by obese v. non-obese.

|  | Younger than 65  (Adipokines Separately)  N=1320  P-Y= 14,776; Events: 126 | | Older than 65  (Adipokines Separately)  N=1207  P-Y=12,764; Events: 102 | |  |
| --- | --- | --- | --- | --- | --- |
|  | *HR (95% CI)* | *p* | *HR (95% CI)* | *p* | *p for interaction* |
| High Adiponectin | 1.02 (0.75, 1.38) | 0.91 | 1.62 (0.94, 2.80) | 0.08 | 0.17 |
|  | *HR (95% CI)* | *p* | *HR (95% CI)* | *p* |  |
| High Leptin | 1.65 (1.03, 2.63) | 0.04 | 1.33 (0.82, 2.16) | 0.25 | 0.77 |
|  | *HR (95% CI)* | *p* | *HR (95% CI)* | *p* |  |
| High FGF-21 | 1.56 (1.03, 2.35) | 0.04 | 1.28 (0.95, 1.73) | 0.11 | 0.47 |
|  |  |  |  |  |  |

All models adjusted for age, age^2^, sex, race, BMI, smoking, history of osteoporosis, history of fracture, prior use of bisphosphonates, DAS28, prednisone use, comorbidity score (RDCI), and date of enrollment.

Note: The RDCI was adjusted down 1 point for those with prevalent fracture.
